# Physical Activity, Screen-Based Sedentary Behavior, and Sleep Duration in Adolescents: Youth Risk Behavior Survey, 2011–2013

**DOI:** 10.5888/pcd13.160245

**Published:** 2016-09-15

**Authors:** Youngdeok Kim, Masataka Umeda, Marc Lochbaum, Steven Stegemeier

**Affiliations:** Author Affiliations: Masataka Umeda, Department of Kinesiology, Health, and Nutrition, University of Texas at San Antonio; Marc Lochbaum, Steven Stegemeier, Department of Kinesiology and Sport Management, Texas Tech University, Lubbock, Texas.

## Abstract

This study examined the concurrent associations of physical activity and screen-based sedentary behavior with sleep duration among adolescents by using data from the national Youth Risk Behavior Survey 2011–2013. Using latent class analysis, we identified 4 latent subgroups of adolescents with various levels of physical activity and screen-based sedentary behavior. The subgroup with high levels of physical activity and low levels of sedentary behavior generally showed greater odds of having sufficient sleep (≥8 hours/night) than the other subgroups. Findings imply that concurrent achievement of a high level of physical activity and low level of screen-based sedentary behavior is necessary to promote sufficient sleep among adolescents.

## Objective

Sleep is a key element in promoting healthy growth and development during adolescence. Evidence is mounting that adolescents with insufficient sleep are more likely to have degraded cognitive functioning and increased daytime sleepiness and fatigue than adolescents with sufficient sleep (1). Physical activity (PA) and screen-based sedentary behavior (SB) are receiving attention as focal points for interventions aimed at improving sleep health because of factors related to each behavior — for PA, body tissue restitution, energy conservation, and temperature downregulation (2,3) and for screen-based SB, displacement of sleep time, arousal (being agitated or “wired”), and light exposure from a screen device (4,5). However, the evidence is largely equivocal (6–8), possibly because of the limited focus on the independent association of each behavior with sleep health; the concurrent associations of those behaviors with sleep health in adolescents are still poorly understood. The objective of this study was to examine the concurrent associations of PA and screen-based SB with sleep duration in a national representative sample of US adolescents.

## Methods

Data came from the Youth Risk Behavior Survey (YRBS), conducted by the Centers for Disease Control and Prevention during the 2011 and 2013 cycles (9). The YRBS is biennial survey with a 3-stage, cluster-sampling design that measures health-risk behaviors among a nationally representative sample of US adolescents in grades 9 through 12 in public and private schools.

We studied 18,253 adolescents who provided valid responses on study variables. Questions on self-reported PA and screen-based SB were used to dichotomize participants into 2 groups (meeting definition or not) for the following 5 items: 1) regular PA (physically active for ≥60 minutes/day during the past 7 days); 2) sports team participation (participation in ≥1 sports teams during the past 12 months); 3) muscle-strengthening exercise (on ≥3 days during the past 7 days); 4) watching television (≥3 hours on an average school day); and 5) playing video or computer games or using a computer for non-school–related work (hereinafter, “video/computer”) (≥3 hours on an average school day). The outcome variable of sleep was based on the self-reported number of sleep hours on an average school night, and participants were dichotomized as either getting sufficient sleep (≥8 hours/night) or not (<8 hours/night).

Latent class analysis (LCA) models were established in a previous study to 1) determine the optimal number of latent subgroups with different response probabilities for PA and screen-based SB items that best fit the data; and 2) examine the associations of latent subgroups with the odds of having sufficient sleep after controlling for potential confounding variables (sex, grade level, fruit and vegetable consumption, no soda consumption, alcohol consumption, tobacco use, marijuana use, depression, sexual intercourse, and obesity) that were selected on the basis of results from a previous study (6). In addition, we used the estimated item-response probability of .50 or more as a threshold to determine the profile of latent subgroups. For example, a subgroup with response probabilities of .50 or more for regular PA, sports team participation, and muscle-strengthening exercise was characterized as having a high level of PA, and a group with response probabilities of .50 or more for watching television and video/computer was characterized as having a high level of screen-based SB. Details on establishing an LCA model using PA and screen-based SB items from the YRBS can be found elsewhere (10).

## Results

The LCA model that had 4 latent subgroups was best fitted to the data compared with the models that had 1, 2, 3, or 5 latent subgroups. On the basis of estimated item-response probabilities on each item, the 4 latent subgroups were characterized as high PA/low SB (30.5%; 95% confidence interval [CI], 28.6%–32.3%), high PA/high SB (12.8%; 95% CI, 11.8%–13.8%), low PA/high SB (39.5%; 95% CI, 37.4%–41.6%), and low PA/low SB (17.2%; 95% CI, 15.8%–18.7%) (Table 1). Most of the high PA/low SB (69.0%) and high PA/high SB (78.6%) groups were male, whereas most of the low PA/low SB group were female (91.4%). The percentage of students characterized as high PA/low SB decreased by grade from 32.9% in grade 9 to 19.9% in grade 12. In contrast, the percentage of students in the low PA/low SB increased by grade from 10.9% in grade 9 to 32.7% in grade 12.

**Table 1:** Descriptive Statistics of 4 Latent Subgroups (N = 18,253), Youth Risk Behavior Survey, 2011–2013^a^

Variable	Total	Latent Subgroup^b^			
		High Level of PA/Low Level of SB	High Level of PA/High Level of SB	Low Level of PA/High Level of SB	Low Level of PA/Low Level of SB
**Overall**	—	30.5 (28.6–32.3)	12.8 (11.8–13.8)	39.5 (37.4–41.6)	17.2 (15.8–18.7)
**Sex**					
Male	49.5 (48.9–50.9)	69.0 (67.2–70.9)	78.6 (76.3–80.9)	42.9 (41.1–44.6)	8.6 (7.0–10.2)
Female	50.5 (49.1–51.9)	31.0 (29.1–32.8)	21.3 (19.1–23.7)	57.1 (55.4–58.9)	91.4 (89.8–93.0)
**Grade level**					
9	26.4 (25.0–27.9)	32.9 (30.1–35.7)	29.5 (26.8–32.2)	27.3 (25.9–28.7)	10.9 (9.2–12.6)
10	25.6 (24.2–26.9)	25.6 (23.2–28.1)	30.6 (28.0–33.3)	22.6 (21.0–24.2)	28.4 (25.7–31.1)
11	24.3 (23.4–25.2)	21.6 (19.8–23.3)	21.6 (20.0–23.2)	25.8 (24.5–27.0)	28.0 (25.6–30.3)
12	23.7 (22.8–24.6)	19.9 (18.5–21.3)	18.3 (16.4–20.1)	24.4 (23.3–25.5)	32.7 (30.2–35.3)
**Eats fruits and vegetables^c^**	21.5 (20.4–22.6)	28.2 (26.3–30.1)	30.9 (28.9–33.0)	15.3 (13.8–16.5)	17.3 (14.9–19.5)
**Does not drink soda^d^**	21.5 (20.3–22.7)	25.2 (23.3–27.2)	15.5 (13.7–17.3)	18.3 (16.7–19.9)	26.9 (24.1–29.7)
**Drinks alcohol^e^**	36.3 (34.8–37.9)	37.0 (34.7–39.3)	39.2 (36.6–41.8)	33.8 (31.9–35.7)	38.8 (35.4–42.2)
**Uses tobacco^f^**	22.9 (21.2–24.5)	25.3 (23.5–27.0)	25.2 (22.5–27.8)	21.0 (19.0–23.1)	21.2 (18.3–24.0)
**Uses marijuana^g^**	13.3 (11.0–15.5)	12.1 (9.5–14.7)	17.1 (14.2–19.9)	14.6 (12.2–16.9)	9.4 (7.0–11.9)
**Is depressed^h^**	28.3 (27.0–29.5)	21.6 (19.8–23.5)	23.7 (21.6–25.9)	34.1 (32.4–35.8)	30.2 (27.5–32.8)
**Has had sexual intercourse^i^**	32.3 (30.5–34.0)	30.3 (28.0–32.5)	35.8 (33.3–38.2)	30.2 (28.3–32.1)	37.8 (34.2–41.4)
**Is obese^j^**	13.0 (12.0–13.9)	7.9 (6.5–9.3)	16.0 (14.3–17.6)	16.9 (15.6–18.2)	9.5 (8.2–10.8)
**Gets sufficient sleep (≥8 h/night)**	31.7 (30.6–32.8)	39.4 (37.7–41.2)	33.9 (31.6–36.3)	24.6 (23.2–26.0)	32.7 (30.2–35.2)
**Item-response probability (95% confidence interval)**					
Engages in regular PA (≥60 min/d)	—	.63 (.53–.71)	.65 (.56–.72)	.05 (.04–.07)	.06 (.05–.08)
Participates on sports team (≥1 team/y)	—	.83 (.79–.87)	.77 (.73–.81)	.32 (.26–.40)	.33 (.27–.39)
Does muscle-strengthening exercise (≥3 d/wk)	—	.90 (.87–.92)	.92 (.89–.94)	.20 (.17–.24)	.27 (.20–.36)
Watches television ≥3 h/d	—	.06 (.05–.08)	.67 (.60–.73)	.58 (.53–.63)	.14 (.11–.18)
Watches video or uses computer ≥3 h/d	—	.14 (.12–.18)	.62 (.53–.69)	.62 (.57–.67)	.10 (.08–.14)

Abbreviations: PA, physical activity; SB, sedentary behavior.

a All values are percentage (95% confidence interval) unless otherwise indicated.

b Latent class analysis with 4 latent subgroups was best fitted relative to the models with 1, 2, 3, and 5 latent subgroups based on fit statistics (ie, lower values for Akaike information criterion, Bayesian information criterion [BIC], sample-size–adjusted BIC, and higher values on average classification probability). Details on methods are available elsewhere (10). The profile of 4 latent subgroups was determined according to the item-response probabilities of 3 PA–related and 2 screen-based SB questions estimated from the conditional latent class analysis controlling for age and grade.

c Ate fruits and vegetables ≥5 times per day during the past 7 days.

d Drank a can, bottle, or glass of soda or pop 0 times per day during the past 7 days.

e Had ≥1 drink of alcohol on ≥1 days of the past 30 days.

f Smoked cigarettes or cigars or used chewing tobacco, snuff, or dip on ≥1 days of the past 30 days.

g Used marijuana ≥1 times during the past 30 days.

h Felt so sad or hopeless almost every day for 2 weeks or more in a row that they stopped doing some usual activities during the past 12 days.

i Had sexual intercourse with ≥1 people during the past 3 months.

j ≥95th percentile for body mass index, by age and sex.

Table 2 presents the conditional LCA that predicts the odds of having sufficient sleep after controlling for confounding variables. Compared with the high PA/low SB subgroup, the low PA/high SB subgroup had lower odds (adjusted odds ratio [AOR] = 0.59; 95% CI, 0.49–0.70) of having sufficient sleep, as did the high PA/high SB subgroup (AOR = 0.76; 95% CI, 0.58–1.00). However, we found no differences in the odds of having sufficient sleep in the low PA/high SB subgroup, low PA/low SB subgroup, or high PA/high SB subgroup.

**Table 2:** Adjusted Odds Ratio of Having Sufficient Sleep (≥8 Hours per Night) Between Latent Subgroups, Youth Risk Behavior Survey, 2011–2013

Comparisons	LogB (SE)	*P* Value	AOR^a^ (95% CI)
**High level of PA/Low level of SB (reference)**			
High level of PA/High level of SB	−0.27 (0.1)	.05	0.76 (0.58–1.00)
Low level of PA/High level of SB	−0.53 (0.1)	<.001	0.59 (0.49–0.70)
Low level of PA/Low level of SB	−0.15 (0.2)	.45	0.87 (0.59–1.26)
**High level of PA/High level of SB (reference)**			
Low level of PA/High level of SB	−0.26 (0.2)	.08	0.77 (0.57–1.04)
Low level of PA/Low level of SB	0.13 (0.2)	.44	1.14 (0.82–1.57)
**Low level of PA/Low level of SB (reference)**			
Low level of PA/High level of SB	0.39 (0.2)	.08	1.48 (0.96–2.27)

Abbreviations: AOR, adjusted odds ratio; CI, confidence interval; PA, physical activity; SB, sedentary behavior; SE, standard error.

a The adjusted odds ratios of having sufficient sleep (≥8 h/night) were estimated from the conditional latent class analysis with a sleep variable as a distal outcome after controlling for sex, grade level, fruit and vegetable consumption, no soda consumption, alcohol consumption, tobacco use, marijuana use, depression, sexual intercourse, and obesity.

## Discussion

Our results generally support the evidence for concurrent associations between PA and screen-based SB and sufficient sleep in adolescents (6,11). In our study, however, the subgroup who concurrently had high levels of PA and low levels of screen-based SB had greater odds of having sufficient sleep when compared with subgroups who had high levels of screen-based SB regardless of their concurrent levels of PA; however, we found no statistical difference in the odds of having sufficient sleep when comparing the subgroup who concurrently had high levels of PA and low levels of screen-based SB with the subgroup who had both low levels of PA and low levels of screen-based SB. Furthermore, when comparing the subgroups with either low levels of PA or high levels of screen-based SB or both, we found no statistical differences in the odds of having sufficient sleep between them. Although the latter results are difficult to interpret, they may indicate that PA and screen-based SB interactively influence sleep duration among adolescents and that improvements in sleep duration can be expected particularly when high levels of PA and low levels of screen-based SB are concurrently achieved.

Our findings should be interpreted cautiously because of the study design, a secondary analysis of cross-sectional data; the use of subjective measures of PA and screen-based SB; and the unexplained measurement errors in classification of the subgroups in the LCA models.

Our study furthers the understanding of the roles of PA and screen-based SB in sleep duration among adolescents by focusing on concurrent associations. The findings emphasize that, in developing future intervention strategies, both behavioral components should be considered concurrently to promote sufficient sleep among adolescents.
